# Feasibility and Acceptability of a “Train the Leader” Model for Disseminating Tai Chi Prime with Fidelity in African American/Black and Latinx Communities: A Pilot Mixed-Methods Implementation Study

**DOI:** 10.3390/healthcare13202622

**Published:** 2025-10-18

**Authors:** Ejura Yetunde Salihu, Kristine Hallisy, Selina Baidoo, Jéssica S. Malta, Cheryl Ferrill, Fabiola Melgoza, Rachel Sandretto, Patricia Corrigan Culotti, Betty Chewning

**Affiliations:** 1 Social and Administrative Sciences Division, School of Pharmacy, University of Wisconsin-Madison, Madison, WI 53705, USA; betty.chewning@wisc.edu; 2 Department of Family Medicine and Community Health, University of Wisconsin-Madison, Madison, WI 53705, USA; hallisy@pt.wisc.edu; 3 Department of Sociology, Western Michigan University, Kalamazoo, MI 49008, USA; sellybaidoo@gmail.com; 4 School of Medicine and Public Health, University of Wisconsin-Madison, Madison, WI 53726, USA; malta.jessicas@gmail.com; 5 Inclusive Tai Chi Prime Community Advisory Board, Milwaukee, WI 53204, USA; cheryferr@gmail.com (C.F.); 12fabimelgoz@gmail.com (F.M.); taichihealth.rs@gmail.com (R.S.); pat@enhancingbalance.com (P.C.C.); 6 Tai Chi Health, LLC, Madison, WI 53711, USA; 7 Enhancing Balance LLC, Brookfield, WI 53005, USA

**Keywords:** treatment fidelity framework, train the leader model, tai chi prime, fall prevention, peer support, African American/black, Latinx, community-engaged research

## Abstract

**Background:** African American (AA)/Black and Latinx communities have limited access to evidence-based fall prevention programs such as Tai Chi Prime (TCP). Community-led interventions that incorporate peer support are cost-effective and sustainable. Using the Treatment Fidelity Framework (TFF) and a mixed-methods research approach, we evaluated the training and support given to trainees during the TCP leader training pathway process and their subsequent fidelity in delivering six culturally tailored community courses. **Methods:** Trainees completed feedback forms after each TCP leader training pathway course. Using a fidelity checklist, a TCP master trainer rated six community TCP classes led by race- and language-concordant leaders. Trainees were invited to participate in virtual one-on-one semi-structured interviews to share their perspectives on the appropriateness and relevance of the TCP leader training pathway and their experience leading community TCP classes. Quantitative data was analyzed using descriptive statistics on Microsoft Excel. Three study team members coded qualitative data using directed content analysis approach. **Results:** Twenty-five candidates enrolled in the TCP leader training. Forty-eight percent identified as AA/Black while 52% identified as Latinx. Eleven trainees (six AA/Black and five Latinx) completed the entire TCP leader training pathway to become certified TCP leaders. Trainees rated the training process as highly satisfactory and appropriate. Leaders from both communities received high fidelity scores for community course delivery. **Conclusions**: Findings contribute to the existing literature, particularly regarding how to effectively disseminate and evaluate a culturally tailored TCP leader training and certification process for culturally diverse communities while maintaining fidelity to the curriculum.

## 1. Introduction

Falls are a pressing public health crisis. Every year, one in four American older adults suffers a fall [1]. Despite an abundance of evidence-based fall prevention programs, participation among older adults in underserved communities of color remains low [2,3,4]. Interventions involving peer support through concordant leaders record stronger metrics of program success (e.g., recruitment, retention and outcomes) compared to programs without peer support [5,6,7,8,9,10,11,12,13]. This finding has been replicated in studies of cancer [12,14,15], diabetes self-management [5,16,17], lupus [18], fall prevention [11,19], and exercise adherence in adolescents [20] and older adults [21]. The effectiveness of peer-led interventions has been linked to the nonhierarchical relationships and equitable power sharing between participants, largely absent in other research and clinical settings [11,16,22].

Tai Chi Prime (TCP), a National Council on Aging-certified fall prevention program, is well-suited for individuals with physical limitations (e.g., leg weakness, gait/mobility and balance issues) [23,24,25,26]. Despite the proven benefits of TCP for balance, leg strength, gait/mobility and executive function [23,24], and the rising rates of falls and fall-related deaths in Wisconsin [1], TCP has not been widely implemented across the state. Delivering programs by representatives of the community can be an efficacious, cost-effective and sustainable way to disseminate evidence-based fall prevention programs, especially in communities of color [11].

Prior studies on how to effectively deliver TC programs in diverse communities have proposed these guidelines: (1) TC programs should be evidence-based and validated by research; (2) they should be delivered by people who have received specific training; and (3) they should be delivered with fidelity, regardless of the local context [27,28,29,30]. TCP already meets the first criterion. The goal of this mixed-methods study [31] is to evaluate how the last two criteria were met and sustained in the Inclusive TCP project using the Treatment Fidelity Framework (TFF) [32].

**Figure 1:**
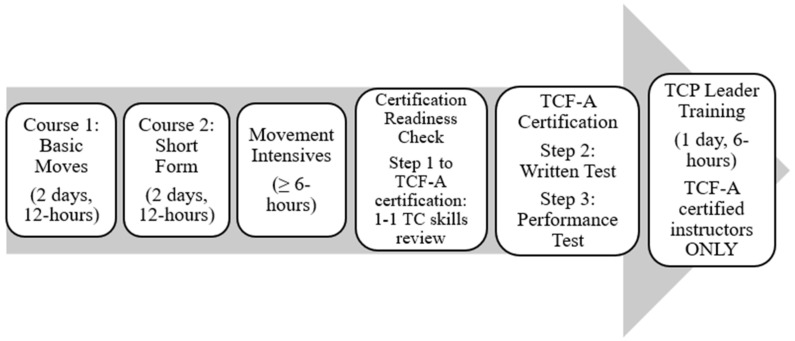
TCP Leader Training Pathway and Certification Process.

The TCP leader training pathway is rigorous and can take 6–12 months to complete. Figure 1 outlines the complete training. Trainees who complete all the steps become certified instructors in both Tai Chi Fundamentals Adapted (TCF-Adapted) program and the evidence-based TCP program (see Figure 1) [25]. This is much more than many community TC programs require of their trainees/certified leaders. Certified TCP leaders are also expected to remain current and authentic to TC by taking 12 h of continuing education credit every two years.

### Treatment Fidelity Framework

To help delineate key components of the training process and outcomes, the Treatment Fidelity Framework (TFF) was used [32]. TFF comprehensively addresses the tension between fidelity and program adaptations, a key metric when implementing health behavior interventions in any population but particularly relevant with our TCP cultural adaptations. TFF has five constructs, Study Design, Provider Training, Treatment Delivery, Treatment Receipt, and Enactment of Treatment Skills.

1. Study Design refers to the planning and design of the study, including the target population, intervention goals, and methods of delivery. For the Inclusive TCP project, a community advisory board (CAB) was used in an iterative, participatory research approach [33].
2. Provider Training refers to how many trainees received pieces of or the entirety of the training program required for TCP leaders (see Figure 1). Within the TFF, the tightly structured and rigorous TCP leader pathway training process (≥36 contact hours, readiness check, written test, performance test) is recognized as essential for the efficacy of the program. The Provider Training construct particularly focuses on whether leaders were adequately trained to deliver the specific TC intervention used in the TCP program (TCF-Adapted program) protocol, skills and teaching strategies. Furthermore, did trainees certified in the TCF-Adapted program complete the TCP leader training portion of the program by taking the final 6 h TCP curriculum course? Resources for this entire Provider Training process include TCP program website, book, DVD (videos) and course handouts. Completion counts and rates were collected for each training step through attendance logs.
3. Treatment Delivery specifically refers to how trainees perceived the delivery of the TCP leader training pathway by the course instructors. Feedback included standardized post-course evaluations after each course, including critique of training resources (website, books, DVDs, course handouts) and learner motivations to complete the ambitious training process and certification. This construct addresses the extent to which leader trainees found the training acceptable and appropriate.
4. Treatment Receipt focuses on trainees’ uptake of the TCP leader training pathway, i.e., did trainees understand and demonstrate the key principles and skills (through the movement and written tests) needed to become a certified TCP leader including cognitive skills, physical skills and behavioral teaching skills. Certification completion rates were collected through attendance logs.
5. Enactment of Skills refers to the newly trained community TCP leaders’ ability to enact the skills they learned during their training while delivering TCP in their respective communities. The five leaders that implemented the six community TCP courses received a fidelity observation by the TCP master trainer. Feedback on adherence to TCP course protocols, the use of specific techniques, and the quality of delivery were rated by the master trainer, and feedback on teaching performance and suggestions for improvement were provided. The fidelity checklist can be found in Appendix A.

## 2. Materials and Methods

### 2.1. Study Design

Using a Community-Based Participatory Research (CBPR) approach [22], researchers formed a community advisory board (CAB) of eight community leaders living in Wisconsin’s most racially diverse city (Milwaukee) to adapt and disseminate the TCP program with fidelity in African American (AA)/Black and Latinx communities. Over the course of two years, the CAB met with researchers to plan and recruit AA/Black and Latinx candidates to take the TCP leader training pathway, adapt the program delivery to meet the cultural needs of both communities, and deliver six culturally tailored community TCP classes (n = 3 in each community) by race-concordant leaders.

A mixed-methods design was employed to comprehensively capture two key outcomes: (1) assessing the acceptability, fit, and feasibility of the TCP leader pathway training program, and (2) evaluating the fidelity of newly trained community TCP leaders to the TCP curriculum [31].

### 2.2. Data Collection

Following the TFF, quantitative and qualitative data were collected from three main sources in addition to attendance logs: (1) TCP post-course feedback forms, (2) interviews with trainees and (3) fidelity checklist ratings by master trainer.

Trainee Attendance and Trainee Feedback were gathered at all TCP leader training pathway courses. Participants were asked to complete standardized 10-item anonymous course feedback questionnaires (See Appendix B) [25]. The feedback forms, which have been used in a prior randomized controlled trial [23], captured quantitative (Likert scale) and qualitative data on course content, delivery, instructor delivery style, and suggestions for improvement. The feedback forms were administered immediately after each training session to ensure timely and accurate responses.

Trainees’ Perspectives on TCP Training:

Thirteen individuals (seven AA/Black and six Latinx trainees) participated in semi-structured one-on-one interviews. A semi-structured interview guide was developed based on TFF by the research team and reviewed by the CAB (see Appendix C). The interview questions explored factors that impacted recruitment and the outcomes of the TCP leader training pathway courses. During the interviews, trainees were asked to share their perspectives on the leader training pathway, and their own subsequent experience leading TCP classes in their respective communities. Trainees also reflected on their future plan to offer TCP in their communities.

Interviews were conducted by an experienced qualitative researcher (E.Y.S). The interviews were conducted either virtually or in-person, depending on participants’ preferences. Interviews lasted 40–60 min. The trainees provided verbal consent for the audio-recording of the interview. The interviews were audiotaped and transcribed by three research assistants. Transcripts were de-identified prior to analysis.

Fidelity Assessment. A TCP master trainer assessed race-concordant leader trainees’ fidelity to the TCP curriculum by observing how they taught the community TCP classes. Fidelity data was collected in the implementation phase of the grant (Year 2, between September 2023 and April 2024). The master trainer observed each of the six community classes once (n = 6). A standardized checklist (see Appendix A) validated in prior TCP research was used. In addition to the numerical rating, qualitative assessment and notes for improvement were collected.

### 2.3. Data Analysis

Using Microsoft Excel, descriptive statistics were used to summarize trainees’ characteristics and their perspectives on the TCP leader training pathway courses and implementation fidelity scores. Open-ended questions at the end of the feedback forms and fidelity checklist were collated and analyzed manually using content analysis [34,35,36,37,38,39].

Three researchers (EYS, SB and JM) analyzed the data independently using directed content analysis [16,35,39,40,41]. Interview data were managed and analyzed using NVivo version 14 [42]. The researchers first read the interview transcripts line-by-line to immerse themselves in the data then independently coded the transcripts. Constructs from the TFF formed the initial categorization matrix and inductive codes were allowed to emerge. All transcripts were independently coded using the codebook. Afterward, the coders consolidated their codes into categories. The categories were then organized into relevant themes [39,43]. The researchers addressed the criteria for trustworthiness in qualitative research by maintaining reflexive memos and audit trails. Multiple coders helped to mitigate the impact of individual bias and enhanced the credibility of findings [38]. Disagreements that persisted after the meetings were resolved by discussing them with other members of the research team [34,35].

Ethical approval was obtained from the University of Wisconsin-Madison Institutional Review Board [#2023-1038].

## 3. Results

This section presents quantitative and qualitative findings organized within four TFF constructs, Provider Training, Treatment Delivery, Treatment Receipt, and Enactment of Skills. The section starts with the Provider Training construct and concludes with an inductive code, TCP Skills Maintenance.

### 3.1. Provider Training

Overall, 25 trainees enrolled in the study, with 14 individuals placed on the waitlist due to limited space and funding. This exceeded the TCP leader training recruitment benchmark of 20. Of the 25 trainees enrolled in the training courses, 48% were AA/Black (8 females, 4 males) and 52% were Latinx (12 females, 1 male). The high recruitment rate recorded was due to efforts of the community partners and CAB members. Three main strategies were employed to recruit candidates for the TCP leader training pathway: word of mouth, website advertisement and social media (Facebook) advertisement. Two community information sessions were held with a TCP master trainer available to answer prospective trainee questions. Word of mouth proved to be the most effective in this study, with teachers of other community exercise programs being the key demographic recruited.

### 3.2. Treatment Delivery

The Treatment Delivery construct refers to how trainees perceived the training they received to become certified TCP leaders. Trainees evaluated various aspects of the course, including course content and delivery, instructor knowledge, and overall satisfaction with the training on a 1–5 Likert scale where 5 was most positive. Ninety-six percent of candidates completed the anonymous post-course feedback surveys (see Table 1).

The Intensive courses are supplemental courses. One movement intensive (6 h) is required by all trainees (see Figure 1) to obtain the 30 h of formal training necessary to be eligible for TCF-Adapted certification. Most successful trainees (11 of 13) took one to two extra movement intensives (the first class had 14 participants while the second class had 15 participants).

Quantitative scores showed high satisfaction with all elements of the training course (i.e., instructional materials, content) across all participants. Qualitative feedback emphasized several key aspects that contributed to their satisfaction. One trainee stated: “I would recommend this course because the instructors are very knowledgeable and kind, the materials are great, and the pace of the course is perfect for learners.”

Trainees also expressed their belief in holistic benefits that TCP can provide for their community with one trainee stating: “[I am] learning how to provide a service that is beneficial to our community mentally, physically, and spiritually.”

### 3.3. Treatment Receipt

The Treatment Receipt construct focuses on trainees’ uptake of the training, including cognitive skills, physical skills and behavioral teaching skills needed to become a TCP class leader. Eleven trainees received all the training and passed both assessments (written and movement tests) required to be certified TCP class leaders in their respective communities (see Table 2). In the table below, retention rate calculation was based on sequential completion of each preceding task to capture the percentage of participants lost at each stage. The highest drop-out rate was observed at the three-step TCF certification stage. This stage includes a one-on-one readiness check, a written test, and a performance test. This is consistent with participants’ interviews, indicating their fear of the testing process was a primary barrier to training completion.

### 3.4. Enactment of Skills (Fidelity to TCP Curriculum)

The Enactment of Skills construct addresses the extent to which the newly certified TCP class leaders delivered the program as intended. This includes fidelity ratings from the master trainer and perspective from the class leaders as gleaned from interviews.

Five TCP class leaders (two AA/Black and three Latinx leaders) led six community TCP classes, three in each of their respective communities. TCP courses were team taught (2 leaders) to provide mutual support. To promote confidence among the newly certified TCP leaders, coaching support from master course instructors was provided on several occasions and gradually diminished over the three TCP community courses. For course 1, coaching was provided 3 times: Day 1 (orientation), Day 2 (Practice Planner implementation) and at the fidelity check. For course 2, coaching was provided 3 times: Day 1 (orientation), Day 4 (Practice Tracker implementation) and at the fidelity check. For course 3, coaching was provided 2 times: Day 1 (orientation) and at the fidelity check.

The classes in the Latinx community were taught completely in Spanish, with course materials that were translated into Spanish by the CAB in the first year of the grant. Classes in AA/Black community were taught in English, with class leaders making minor modifications depending on the group. Some of the changes in the AA/Black classes include: (1) use of Afro jazz music as background music while practicing the moves and during teatime and (2) use of culturally sensitive examples to explain moves and benefits of TCP.

Using a Likert scale (5 was highest and 1 was lowest fidelity score), the master trainer evaluated general course logistics, course content (warmups and Basic Moves, seated activities, home practice activities, Short Form, closing and instructor teaching skills). The average fidelity score reported was 4.60 in the AA/Black community and 4.65 in the Latinx community. Overall, these ratings by the master trainer indicate high fidelity to TCP by both AA/Black and Latinx class leaders.

Results include feedback from the master trainer on class leaders’ ability to enact the skills they learned (fidelity to TCP curriculum) and interviews with trainees. For the interviews, source is cited as master trainer (MT) or trainee (T1) to designate the person interviewed. The trainee’s race and current stage in the certification process at the time of the interview are also stated at the end of the quote, e.g., AA/Black, certified or Latinx, certified.

Theme 1: Master Trainer’s Qualitative Feedback on Fidelity to TCP’s Core Components.

Qualitative feedback from the master trainer highlighted several key observations from the classes:

“Both [AA/Black class leaders] have wonderful teaching skills and great relationships with participants. They use appropriate language, imagery, and cues that participants understand and seem to enjoy. Both with consistent training and possibly learning the traditional CMC form would be fabulous master trainers for their community.”

—MT

“Both leaders [Latinx class leaders] have great compassion for the participants… Both have great command of Basic Moves and descriptions. They give good reminders and corrections.”

—MT

The master trainer also noted areas for improvement for the class leaders in both communities:

“Areas for improvements include a few more reminders about base structure (shoulder width, upright body) could be added. The timing of this [AA/Black] class may be hindering participant consistency and their ability to stay for a whole class.”

—MT

“Both leaders are doing a great job! Due to my lack of understanding of Spanish, I was not certain all topics were covered but after discussing with them, we determined they were.”

—MT

Theme 2: Trainees’ Experience Enacting TCP Skills in their Communities.

Certified leaders who had led at least one community class by the time of the interview were asked about their experience enacting the skills they learned during training.

#### 3.4.1. Subtheme 1: Balancing Fidelity with Adaptive Structure of TCP

The leaders mentioned that the adaptive nature of the program made it easy to maintain fidelity while still adjusting their delivery to suit their community members’ needs. One leader recounted an experience while teaching her second community TCP class:

“…a lady came with her ankle broken, so she had a boot and couldn’t move, and she was seated, so I taught standing up, and P5 [co-class leader] was teaching her seated, and then when we switched, P5 did the second part standing, I was doing the seated [version]. It was nice having her there, and other people see how we can have people stand or be seated and then adjust their movement.”

—T4 (Latinx, certified)

#### 3.4.2. Subtheme 2: TCP Curriculum Enhances Fidelity Compared to Other Exercise Programs

Some leaders mentioned how having the curriculum made it easier to deliver tailored classes compared to other exercise programs they led in the past.

“It’s unbelievable how you guys have structured the Tai Chi training. That’s one of those things I really love…when I teach yoga, I have to create my own classes. TCP give us the curriculum that we are going to teach every single class in the program. That is great because I don’t have to spend time thinking about how I will show them the next step. I really love it.”

—T2 (Latinx, certified)

“We have the outline for each class, so that’s helpful. The rest is up to the individual instructor to familiarize themselves with the process, what we’re teaching and the way to teach it in the most effective way for the students we are teaching.”

—T12 (AA/Black, certified)

### 3.5. TCP Skills Maintenance

The “Skills Maintenance” construct addresses the potential for TCP to be sustained in these communities after study completion, i.e., the extent to which TCP leaders can maintain their certification and continue teaching TCP in their communities long-term.

Tai Chi Health, LLC (TCP purveyor) and the research grant supported attendance of a biennial three-day TCF retreat for certified instructors. The intention of the retreat is to help attendees improve their tai chi skills and obtain the necessary 12 h of contact training to retain certification for the next two years. Funding TCP leaders’ attendance ensures that newly certified TCP class leaders maintain their certification and remain engaged and proficient in their TC practice which is crucial for TCP fidelity and sustainability. Also, with these new TCP class leaders having the required certification to continue offering classes for two years, the communities served by these leaders will continue to enjoy the health benefits of TCP. This approach to ensuring TCP maintenance not only reinforces TCP’s long-term impact but also its reach in these communities.

Theme 1: Trainees’ Sustained Interest in Leading Community Classes.

Trainees expressed a strong commitment to continuing to lead community TCP classes in different settings. One trainee shared his vision of delivering TCP in schools to help young kids with behavioral issues and academic challenges.

“I see a way to adapt this for the younger kids and help them deal with behavioral issues and teach them some kind of discipline and how to sit still because I think that something like this could help the at-risk youth and poor kids of color…I want to do this for my kids’ school. They don’t have extracurriculars, so I want to bring this to the school’s attention.”

—T10 (AA/Black, certified)

Another trainee was hopeful that once she gets her certification, she can start teaching TCP in her church:

“I could open it up at church right after ladies Bible class, and then we could do the first 10 moves or we could start out learning the first five moves, six moves or whatever, just to start it out. I would like to be able to have an opportunity to do that once I’ve passed my movement test.”

—T7 (AA/Black, trainee)

Trainees also emphasized the importance of continuous learning even while teaching to ensure they stay current on the TCP curriculum. One trainee shared:

“I really like it. Even though I’m teaching, I am still learning. I keep on finding my vibe and adjusting and being conscious about my movements, but I like interacting with people and listening to them [my class participants] and their needs. I want to learn more how to help them in the class.”

—T4 (Latinx, certified)

## 4. Discussion

In this mixed methods study, TFF focused on the leader pathway training program as an important element in strengthening TCP leaders’ self-efficacy and ensuring they have the skills to deliver the program with integrity. The TFF model was useful in achieving the program goal of assessing fit, feasibility and cultural adaptability to the TCP “train-the-leader” model.

To ensure the training was appropriate and effective for these communities, two trainees (one from each of the target communities) were selected as representatives of their cohort to attend monthly CAB meetings, alongside their master trainer. This co-planning and decision-making approach meant that the cohort was kept abreast of research goals early in the process and had the opportunity to share their opinions on their training throughout the TCP leader training process. This student-teacher collaborative approach has been used in education research with remarkable results. Studies that employed this collaborative approach to teaching had better student outcomes, knowledge acquisition and retention, and skill confidence [44,45,46]. We noted similar results in this study. Instead of being passive recipients of TCP education, the trainees were active partners who were able to shape how their training was packaged and delivered in real-time. This approach to learning might have contributed to the high training completion rate. Likewise, leaders displayed high confidence in their skills and eagerness to continue teaching TCP in their respective communities.

In line with recommendations that traditional advertisements are inadequate for addressing the disparity in recruitment and retention of AA/Black and Latinx populations in health promotion research, this study employed a more community-driven approach to recruiting trainees [33,34]. By engaging community-based organizations to plan, recruit, and train individuals from the communities, it allowed for an organic advertising approach to emerge. Most of the trainees recruited for the TCP leader training pathway courses were recruited in person at community events led by members of their own community. By engaging respected community leaders in the recruitment, TCP gained legitimacy in these communities, which facilitated TCP class leaders’ recruitment and retention.

Similarly to previous studies that compared recruitment rates for health promotion research between men and women, recruitment rates for male trainees were lower (n = 5/25 (20%). However, it is worth noting that four of the five male trainees (80%) successfully completed the TCP leader training pathway. The lower recruitment rate among men is consistent with literature that shows that men from racial minorities, especially AA/Black men, are less likely to enroll in health interventions compared to women and their White male counterparts [8]. This result highlights the need to intensify efforts to recruit men from communities of color into health promotion programs through targeted outreach strategies such as partnering with barbershops, gyms, faith-based organizations, and community events.

The high fidelity scores reported for both communities were attributed to the high level of support provided by the research and TCP training team. Aside from the rigorous in-person training courses, trainees had access to additional resources such as online training videos, DVDs, books and course handouts, which supported their home practice. TCP Practice Planners and Trackers, i.e., the tools modeled and utilized in community TCP courses to facilitate home practice habits in community participants, likewise enhanced leader practice habits.

While the master trainer rated the Latinx class leaders highly for their fidelity to the TCP curriculum, she mentioned her lack of language fluency as a limitation in the fidelity check process. This language barrier highlights the need for race-and language-concordant trainers and fidelity reviewers in TCP. It also highlights the need to continue building capacity for the currently certified class leaders to grow into master trainers who can train and evaluate new Spanish speaking TCP class leaders in their community.

Common areas of improvement for class leaders for both communities included (1) better time management to address all elements of the curriculum, (2) more practice on the short form (choreographed dance of TC) and (3) more time allotted to practicing qigong in class. This result highlights the need for class leaders to continue offering TCP in their communities to hone their tai chi and TCP teaching skills.

Overall, the fidelity scores and areas of improvement were similar across both communities, which affirmed that the decision to train both communities together instead of separately was the right choice. Community classes delivered in Spanish with newly translated Spanish materials and handouts ensured cultural appropriateness without compromising fidelity.

A key factor worth mentioning is that all the trainees in this study received their training from the same master trainers throughout the training courses and had the opportunity to engage with their master trainer and receive feedback frequently long before they led their first community class. Likewise, a Spanish speaking TCF-Adapted trainer was available at all times to assist the bilingual trainees throughout the process. The high level of engagement with the master trainers, a co-planning approach used to facilitate learning and the opportunity to receive personalized feedback in the readiness check session (a compulsory component of the training and certification process) might explain the similar fidelity scores across both communities.

TFF was instrumental in ensuring that all aspects of the World Falls Guidelines [28,30] were thoroughly addressed in this study. TFF along with train-the-leader model was useful in ensuring an effective and sustainable dissemination of TCP in underserved communities of color. Future studies should similarly consider adopting this approach to enhance the development and scalability of fall prevention programs, such as TCP, across diverse cultural settings. Additional study outcomes include producing a Latinx version of the TCP program (TCP Vital), which can be used in Latinx communities across the country and/or internationally. Community-facing materials that have been adapted by the CAB for both African American/Black and Latinx communities can be scaled up for use in other underserved populations.

Andersen’s Model of Health Service Utilization [47] offers an additional model for characterizing this work as increasing access to evidence-based health promotion programs in underserved communities. Training community members to lead TCP classes in their communities was key in the success of the project, highlighting the need for more race- and language-congruent leaders and materials to increase access to similar interventions. This model can help researchers and practitioners tailor interventions to meet the unique needs of broader populations, beyond the typical contexts in which such programs are often offered while maintaining fidelity. By doing so, the scalability and cultural adaptability of programs like TCP can be significantly improved. Given the growing pandemic of falls among older adults, findings can support policy efforts to fund fall prevention programs and supportive group exercises. An ongoing study by the research team is evaluating the impact of the culturally adapted TCP program on fall risk and biopsychosocial outcomes in AA/Black and Latinx communities.

### Limitations

Eleven out of the 25 enrolled participants had completed the entire leader pathway training to become TCP class leaders at the time of writing this manuscript. Three more trainees are eligible to take the final 6 h prime leader course. These trainees are still in the pipeline to become TCP class leaders. Primary barriers to training completion included language barrier for Spanish-speaking trainees, anxiety about the written and performance tests, and competing demands on time. Additionally, most of the trainees who were interviewed were those who had already completed the required training hours to become certified leaders, which may introduce a selection bias and impact generalizability. The experiences and reflections shared in the interviews may differ from those who enrolled in the TCP leader training pathway but did not complete the training and those who chose not to participate. This could skew the findings toward more positive experiences. Attempts were made to interview trainees who did not complete the training hours to understand the barriers to retention but due to logistical barriers, we were unable to do so. To mitigate this, we provide results from the feedback forms collected after each course offering, including the supplemental courses (Movement Intensives). Results from these post-course feedback forms also support the high acceptability noted by the trainees who were interviewed. Unfortunately, we could not gather feedback from individuals who chose not to enroll in the training. Despite efforts to follow up with individuals who were personally contacted by community leaders or recruiters but chose not to participate, these attempts were unsuccessful. Also, due to the highly collaborative and community-embedded study design, the relationship between researchers, master trainers, and trainees may have influenced responses. The academic-community partnership was built on open and honest dialogue, which was sustained throughout the study, but the possibility of social desirability bias remains.

Future studies could build on these findings with larger, more diverse samples and explore long-term outcomes of race-concordant TCP delivery in communities of color. The language barrier identified by the master trainer when evaluating Spanish-speaking TCP leaders is a limitation in the fidelity assessment. This barrier highlights the need for bilingual fidelity evaluators in future TCP studies. It is worth noting that the master trainer has been teaching Tai Chi for 40 years and certifying Tai Chi instructors for over 25 years, which lends considerable credence to the evaluation despite this limitation. Lastly, the study was conducted in one Midwestern city in the U.S.; however, TFF is applicable in a global context for addressing the planning and implementation of health behavior training programs. Despite these limitations, this study offers unique insights into how a theory-driven, community-led approach could improve the uptake and sustainability of evidence-based fall prevention programs in communities of color through race-concordant leaders.

## 5. Conclusions

Participatory planning contributed greatly to the successful recruitment and training of race-concordant TCP class leaders from AA/Black and Latinx communities. High recruitment, retention, and course satisfaction rates among trainees reflect their strong interest in TCP. The high fidelity observed in this study exemplifies that the cultural adaptations made by the CAB supported rather than compromised the integrity of TCP core components. The delivery of TCP by race- and language-concordant leaders, supported by a well-intentioned CBPR model, makes for the sustainable dissemination of TCP in AA/Black and Latinx communities.

## Figures and Tables

**Table 1:** Feedback on TCP Leader training pathway courses.

Course Satisfaction (Return/Participant Rates)	Course 1 (25/26)	Course 2 (20/20)	Intensive 1 & 2 (29/30)	Prime Leader Course (8/11)
Content adds value to practice	4.80	4.96	5	*
Content matched written description	4.88	4.95	5	4.86
Instructional methods were appropriate for content	4.88	5	5	5
Handouts added value	4.88	4.95	5	4.71
Session met my expectations	4.80	4.96	5	4.86

* Respondents cannot judge this aspect until they teach.

**Table 2:** TCP leader certification Rates.

Training Stage	AA/Black	Latinx	Total (N)
Enrolled in leader pathway training	n = 12 F (8), M (4)	n = 13 F (12), M (1)	N = 25 *
Completed the required 30+ hours of TCF-A	n = 9 F (5), M (4)	n = 10 F (9), M (1)	N = 19 out of 25 (73%)
Completed TCF-A certification process	n = 8 F (5), M (3)	n = 5 F (4), M (1)	N = 13 out of 19 (68.3%)
Completed TCP Leader Training ^ψ^	n = 6 F (4), M (2)	n = 5 F (4), M (1)	N = 11 out of 13 (84.6%)

KEY: F = female, M = male. Community tai chi programs tend to ‘appeal’ to more females than males. TCF-A = Tai Chi Fundamentals Adapted Program with Optional Side Support (TC form used in TCP). * N = 25 TCP trainee enrollment exceeded expectations (Goal was N = 20). Waitlist (N = 14) was due to funding/space limitations. ^ψ^ TCP Leader Training met grant expectations (Goal was N = 5 per community).

## Data Availability

The data presented in this study are available on request from the corresponding author due to privacy and confidentiality considerations involving participant information.

## Abbreviations

The following abbreviations are used in this manuscript:

TCP	Tai Chi Prime
CAB	Community Advisory Board
TFF	Treatment Fidelity Framework
AA/Black	African American/Black

## Appendix C

**Table:**

Interview Guide for Leader Trainees
Introduction
Thank you for agreeing to be interviewed today. My name is ____. I am a graduate student at the University of Wisconsin-Madison School of Pharmacy. Today we will discuss your experiences in the Tai Chi Prime (TCP) leader training course. Later, we will discuss the community classes you will lead in the Fall. We would like your thoughts on how we can best prepare you to succeed as a TCP leader, so there are no right or wrong answers. Before we begin, do you have any questions you would like to ask about the project? I want to confirm your consent to participate in this research. Is that all right? If it is OK with you, I will record our discussion today, so I do not miss any important comments. All your comments will be strictly confidential, and if we write any reports or publications about this study, we will not use your name. Would that be all right with you?
Introduction:
Can you share a little bit about yourself and your background? Recruitment (5 minutes)
1. Where were you recruited for this project? Follow up: Who recruited you, and how did they introduce the subject?
2. How should we recruit? What information would you have liked to receive?
3. What key qualities or characteristics should we prioritize when recruiting new TCP leaders?
4. What was your motivation to take this training to be a TCP leader?
5. While taking these courses, how did the training help you with your personal and professional goals? Probe: Do you recall teaching people in your community what you have learned so far?
Experiences with training courses (courses 1, 2, and movement intensives) (15 minutes)
6. Please describe in one word your experience in the training courses
7. Based on what you just said as a single word to describe the training, can you elaborate on your experience?
8. What are some of the challenges you experienced with attending the classes? Probe: location of the sessions or timing of the sessions
9. You learned the basic moves of tai chi in course 1. What were your thoughts on the course content and delivery?
10. In Course 2, you learned the short form. What were your thoughts on that session’s content and delivery?
11. What part of the training did you like the most?
12. What part of the training did you like least? Probe: What would have helped you most?
Experience with Written Test
13. Have you taken the written test? If yes, what are your thoughts on the written test? IF NOT, SKIP TO QUESTION 17.
14. What challenges did you encounter while preparing for the test?
15. What strategies did you use to prepare and pass the test?
16. What resources/support would you have liked to receive from the research team to help you prepare?
17. If not, what are your current challenges as you prepare for the test?
Movement test
18. Have you taken the movement test? If yes, what are your thoughts on the test? IF NOT, SKIP TO QUESTION 22.
19. What challenges did you encounter while preparing for the test?
20. What strategies did you use to prepare and pass the test?
21. What resources/support would you have liked to receive from the research team to help you prepare?
22. If not, what are your current challenges as you prepare for the test?
Experiences with Prime Leader Training (5 minutes)
23. Have you taken the Prime Leader training session? If yes, what are your thoughts on the session? IF NOT, SKIP TO QUESTION 20
24. What are some of the challenges you experienced with attending the session? Probe: location of the sessions, delivery format, and timing of the sessions
25. What part of the training did you like the most?
26. What part of the training did you like least?
Perspectives on leading future community classes (5 minutes)
27. How likely are you to offer tai chi in your community within the next six months? If high, why? If low, why not?
28. To what extent do you feel prepared to lead the community classes after taking the classes and the Prime Leader training?
29. How can we encourage African Americans (or Latinx) to be interested in the community classes? What would it take to move this from interest to actively participating?
CLOSING QUESTION
Thank you very much for sharing your thoughts and experiences. Before we close, is there anything else you want to share about your experience in the training courses, written tests, and prime leader training? Do you have any suggestions for future community classes? If you have any questions after this session, please contact me at ________.
